# Supplementary material for: Inhibitory Effects of Tangeretin, a Citrus Peel-Derived Flavonoid, on Breast Cancer Stem Cell Formation through Suppression of Stat3 Signaling
Source: Molecules. 2020 Jun 3;25(11):2599. doi: 10.3390/molecules25112599 (PMC7321155; doi:10.3390/molecules25112599)
Supplement: Supplementary file 1 [file molecules-25-02599-s001.pdf]

Table S1. Specific Real-time RT-qPCR primer sequences containing *nanog, Sox2, Oct4, and β-actin* genes

Genes	Primers
Nanog	Forward: 5′-ATGCCTCACACGGAGACTGT-3′<br>Reverse: 5′-AAGTGGGTTGTTTGCCTTTG-3′
Sox2	Forward: 5′-TTGCTGCCTCTTTAAGACTAGGA-3′<br>Reverse: 5′-CTGGGGCTCAAACTTCTCTC-3′
Oct4	Forward: 5′-AGCAAAACCCGGAGGAGT-3′<br>Reverse: 5′-CCACATCGGCCTGTGTATATC-3′
β-actin	Forward: 5’-TGTTACCAACTGGGACGACA-3’<br>Reverse: 5’-GGGGTGTTGAAGGTCTCAAA-3

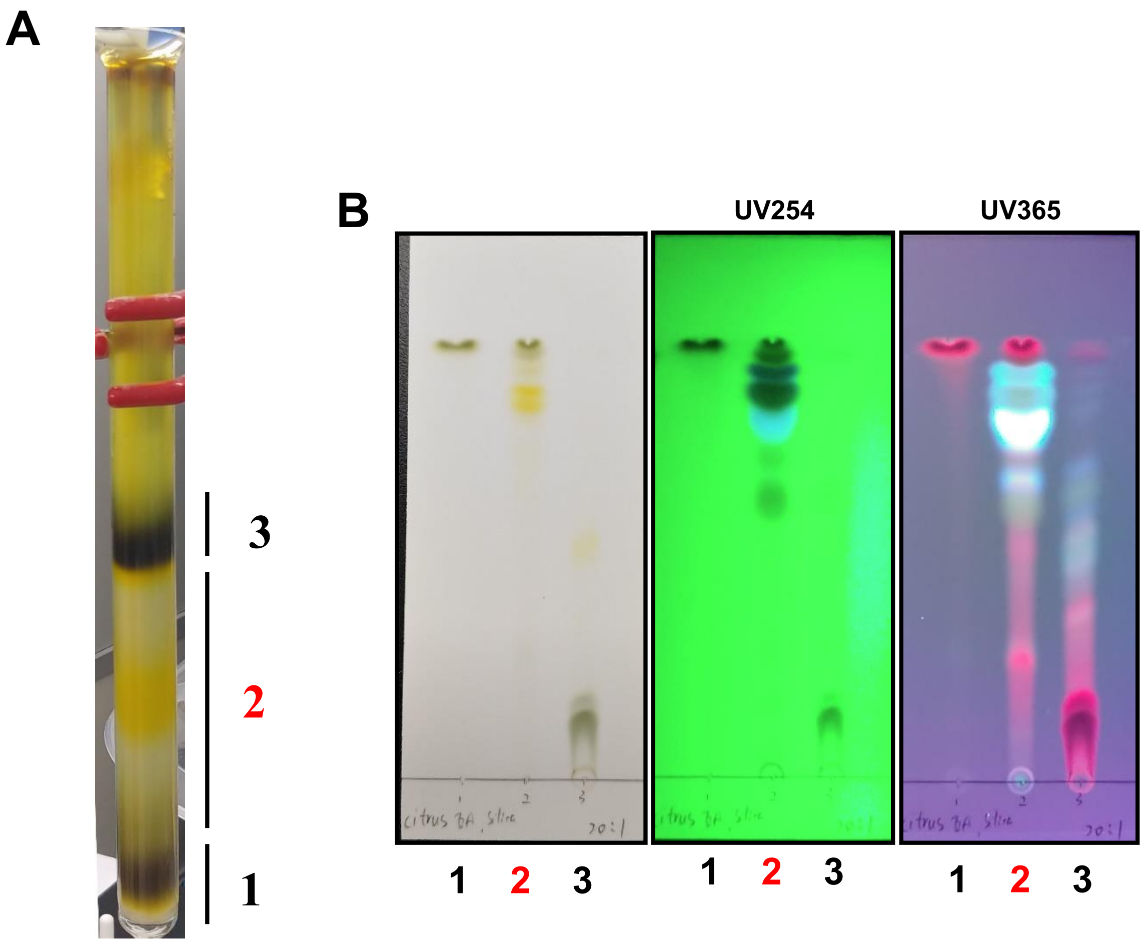

Supplementary Figure S1. Purification of CSC inhibitor derived from citrus extracts using SiO_2 gel chromatography eluted with $CHCl_3$: MeOH (20:1)

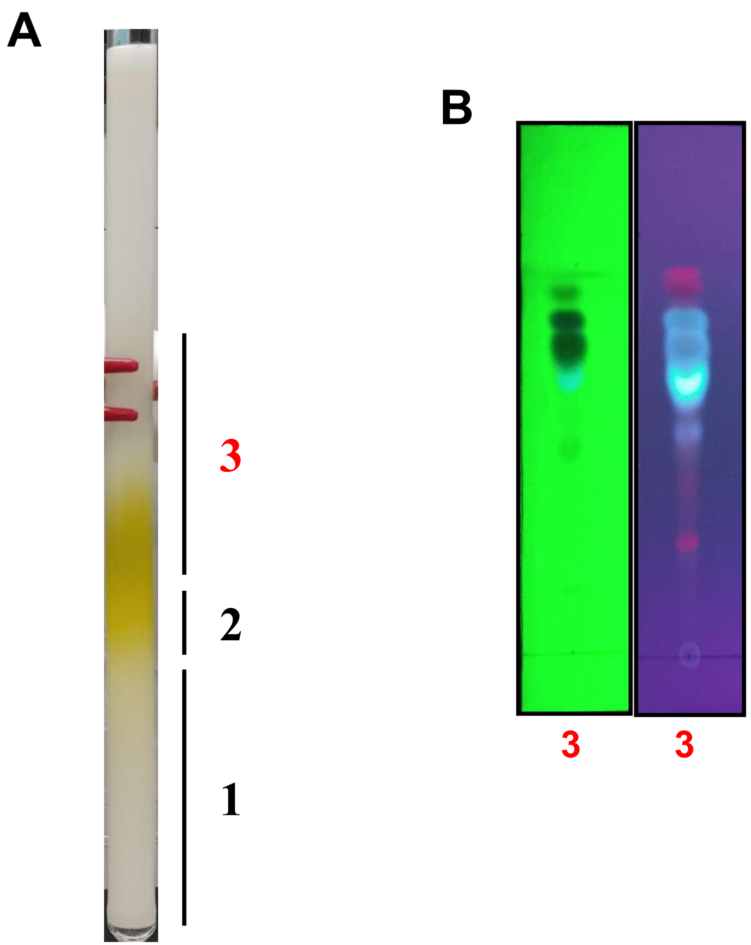

Supplementary Figure S2. Purification of CSC inhibitor from citrus extracts using sephadex LH-20 gel chromatography eluted with MeOH

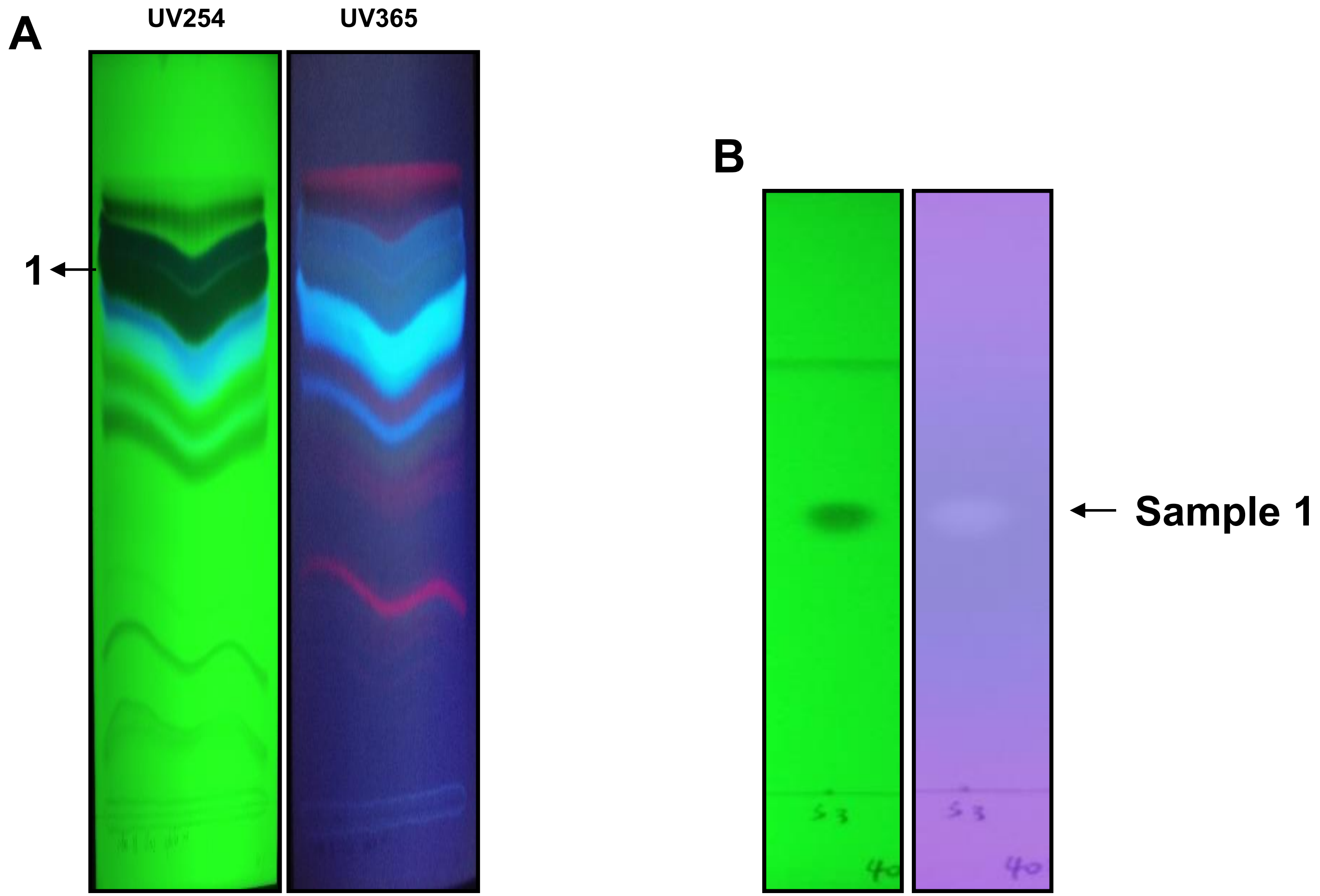

Supplementary Figure S3. Purification of CSC inhibitor from citrus extracts using preparative thin layer chromatography with $CHCl_3$:MeOH (20:1)

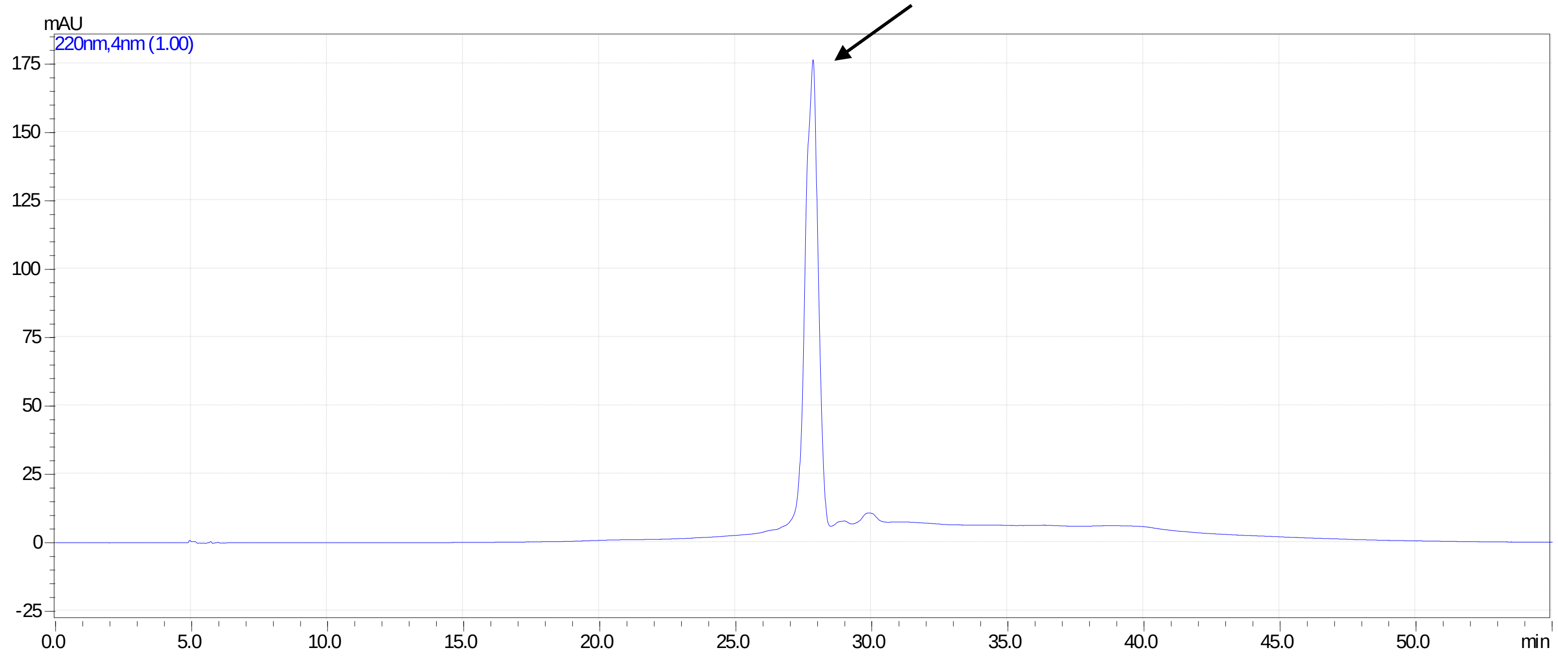

Supplementary Figure S4. Purification of CSC inhibitor using HPLC

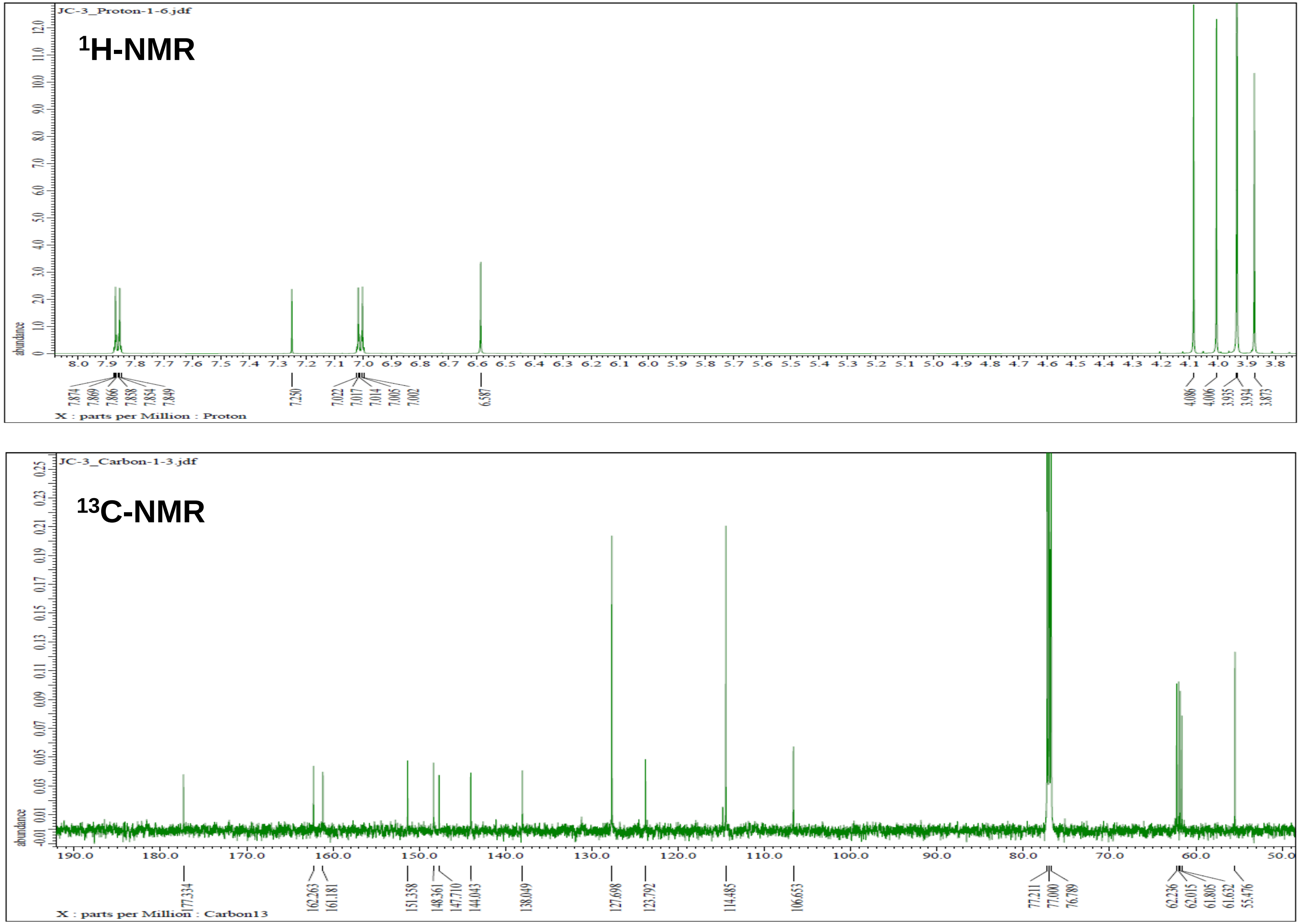

Supplementary Figure S5. ^{1}H NMR and ^{13}C NMR spectra of the purified compound

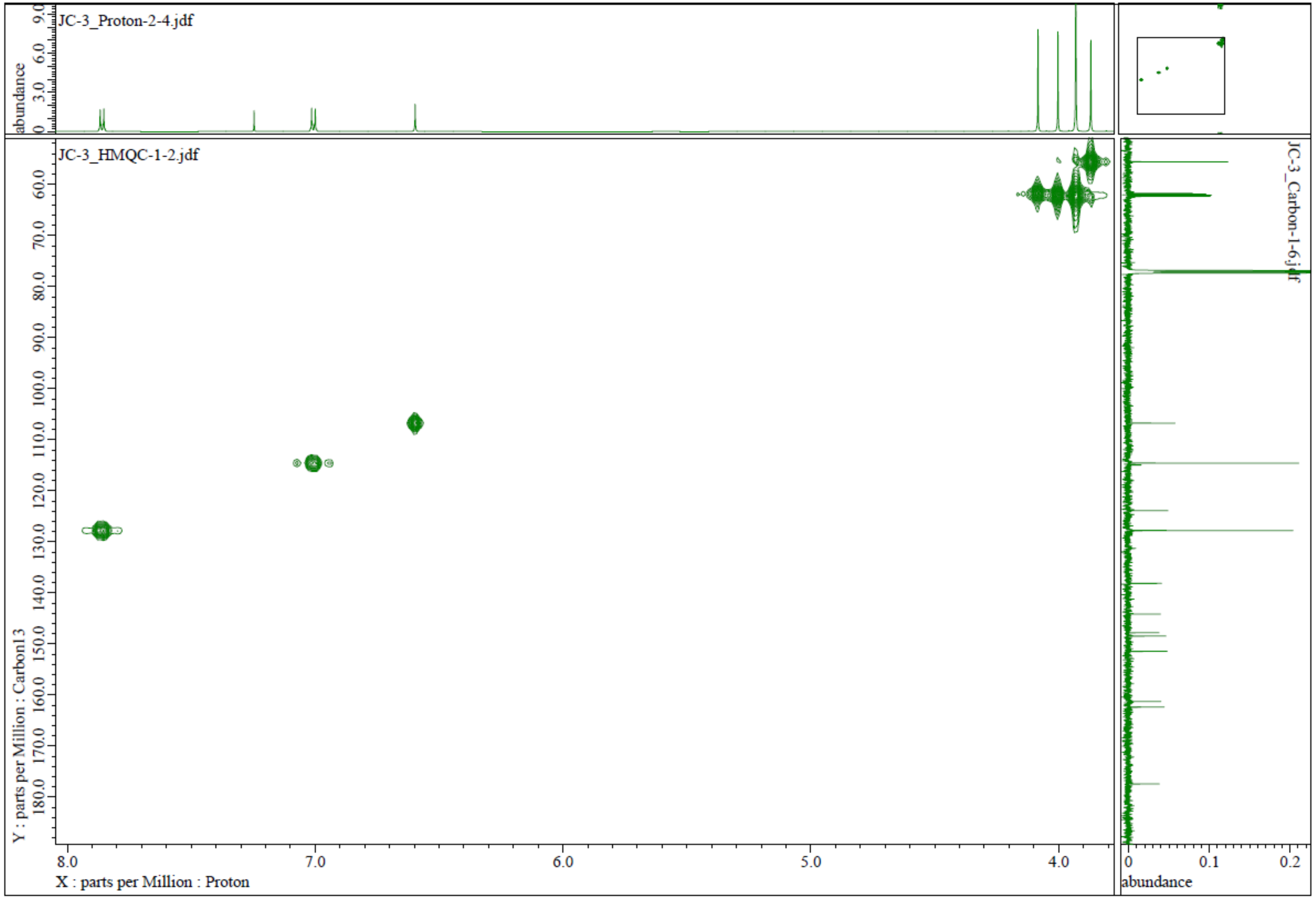

Supplementary Figure S6. HMQC spectrum of the purified compound

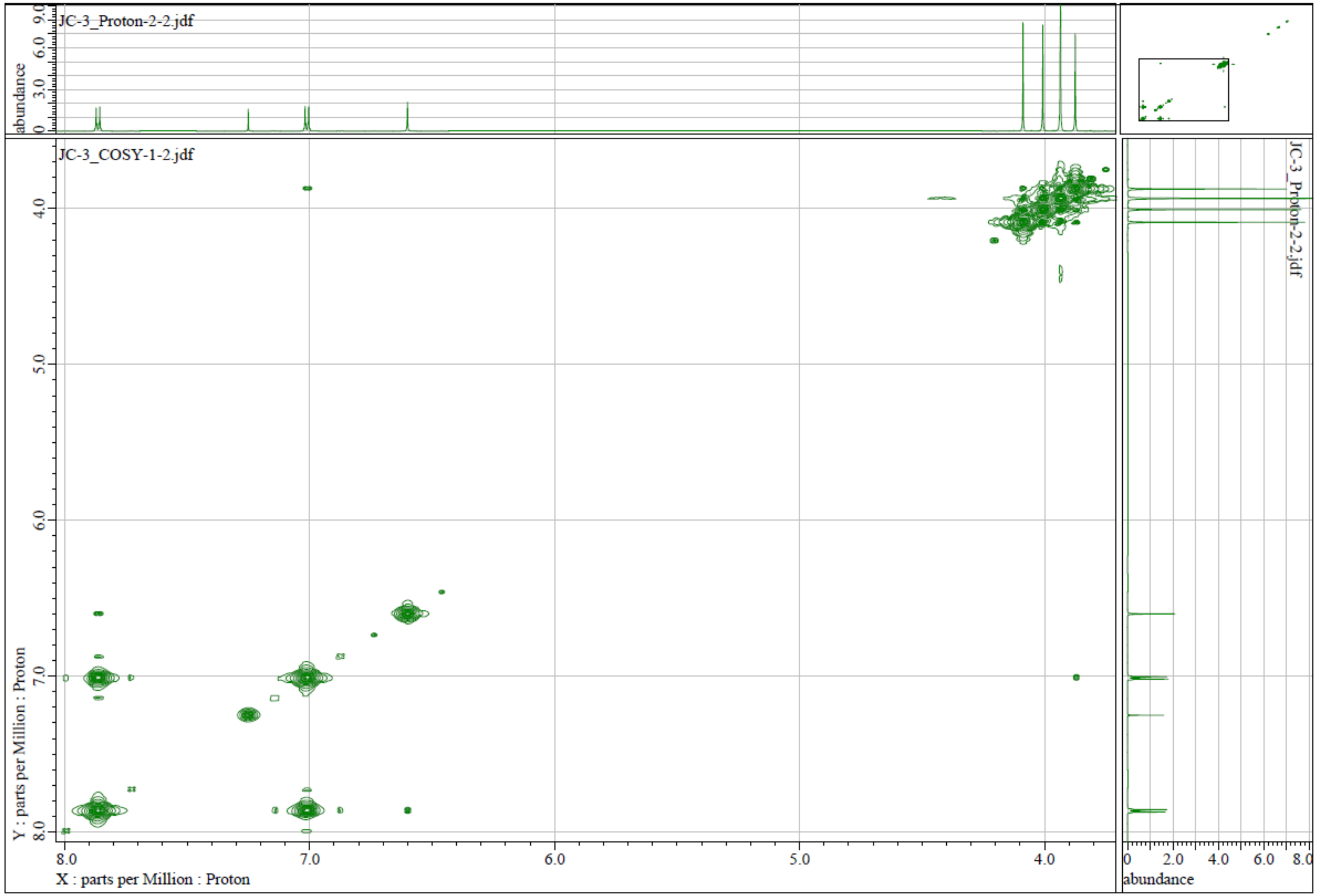

Supplementary Figure S7. ^1H-^1H COSY spectrum of the purified compound

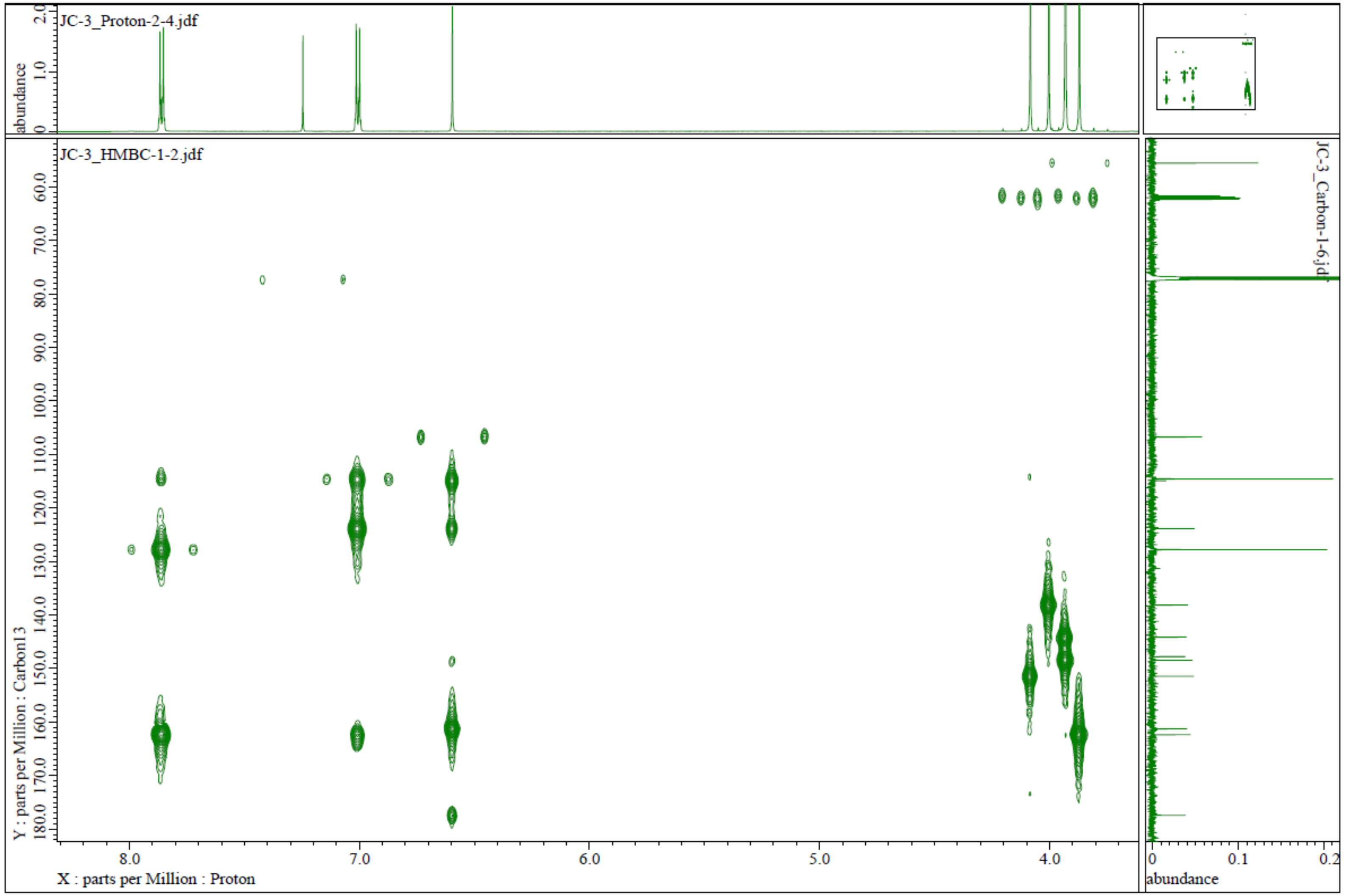

Supplementary Figure S8. HMBC spectrum of the purified compound

A

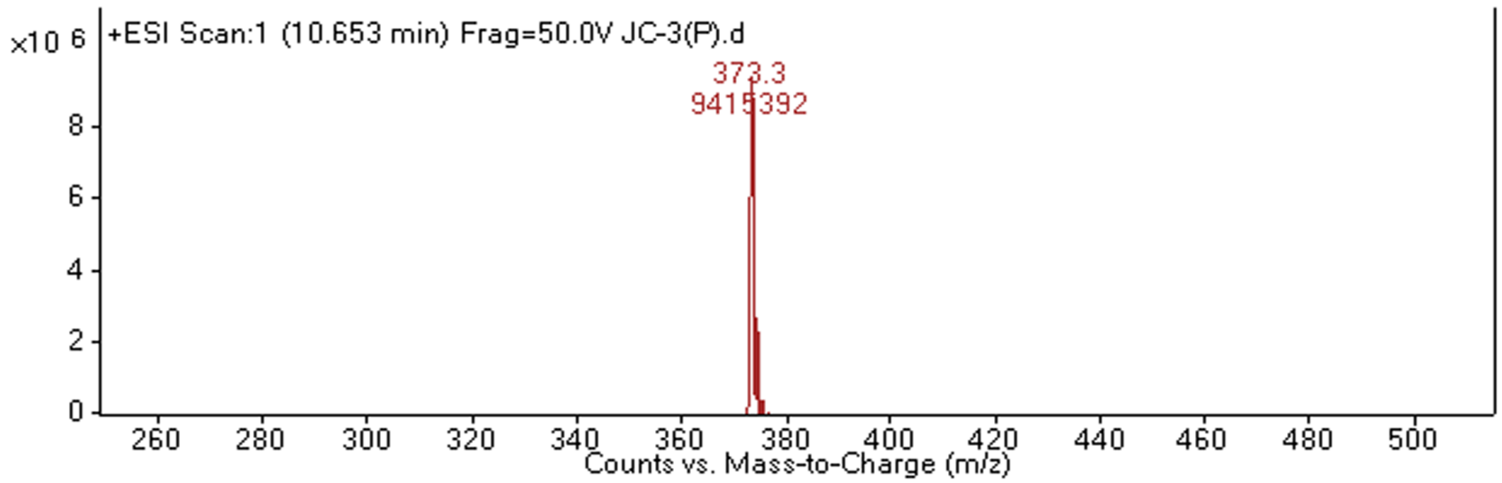

B

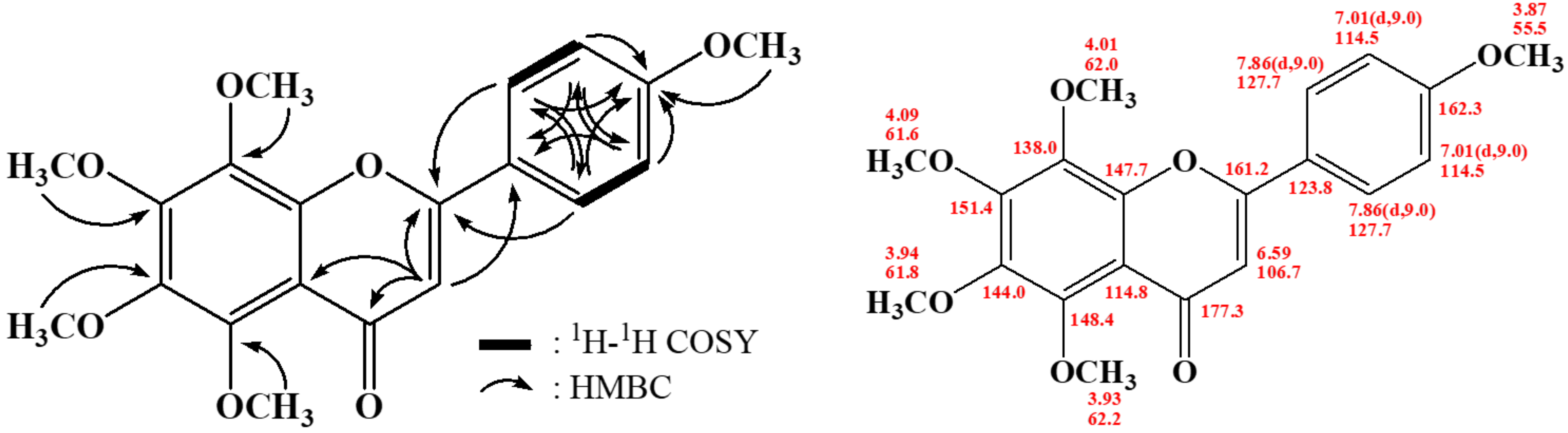

Supplementary Figure S9. ESI mass spectrometry (A) and two-dimensional NMR data and ^{1}H, ^{13}C peaks assignments (B)
